# Alcohol in Materia Medica

**Published:** 1834

**Authors:** Edwin A. At Lee

**Affiliations:** Philadelphia


### Art. II.—Alcohol in the Materia Medica.

*Remarks on the question "Is Alcohol an indispensable article of the Materia Medica."* By Edwin A. At Lee, M. D.

The praiseworthy efforts of the patriotic, the moral, and the pious of our country, for the extinction of *Drunkenness* and its host of evils, have given importance to this question, claiming the serious, deliberate consideration of Practitioners of the healing art, who are at all times ready to co-operate with their fellow citizens, for the promotion and establishment of good order, and national happiness: our object, professionally, is the preservation of "*a sound mind in a sound body.*" And in order that the general movement of the phalanx, or rather army, of *Temperance*, may be harmoniously onward, it is desirable that *our* views and practices on this subject may coincide, so that we may walk by the same rule, and thus our opinions, when either privately or publicly advanced, may tend in some degree to obtund the shaft of sarcasm—which has been *too little* blunted by time:—

—"*Who shall decide when Doctors disagree?*"

It appears that many Physicians of reputation have arrived at the conclusion, and consistently express their sentiment

*Calomel has no tendency to act as a purge in this disease until the secretion of bile is restored.

without qualification, that *Alcohol*, either alone, or in any of its modifications, is altogether needless, in a remedial view; and even that it has been much more injurious than beneficial.

A considerable number, doubtless equally respectable with those just mentioned, are yet unprepared, either from theory or experience, to commit themselves in so unqualified a manner, being confident, that in their practice, alcoholic preparations have been signally useful in a variety of diseases.

I acknowledge myself to be too much inclined to the sentiment of the latter class of Practitioners, and on this account am desirous of eliciting the opinions of my brethren, who may convince me of the existence of substitutes hitherto beyond the limits of my knowledge, that may answer all the indications of treatment, in which recourse has generally been had to the article in question, and that may, in the hands of Physicians much more judicious than myself, have had more prompt and happy effects.

From the sensible qualities of alcoholic preparations, we would, *a priori*, be of the judgment, that they ought to be potent remedial agents, especially in diseases of an *asthenic* type. Experience seems to have fully sanctioned and confirmed such judgment, since the pharmaceutical uses of Ethers, Tinctures, Extracts, Aromatic sulphuric Acids, &c. &c. are neither few nor of recent origin.

It is no valid objection to the proper use of this article, that the excessive use, even with reference to its therapeutic agency, has proved deleterious: the same objection lies against the administration of a great number of valuable remedies, the virtues of which are sufficiently tested and approved.

That we may the better determine relative to the merits of Alcohol, let us, without analysing it chemically, examine its medical properties, as set forth in the U. S. Dispensatory; viz: "Acohol is a very powerful stimulant. It is the intoxicating ingredient in all spirituous and vinous liquors, including under the latter term, porter, ale, and cider, and every liquid in short which has undergone the vinous fermentation. In its pure state it is never used in medicine, but diluted to various degrees, it forms the menstruum for many remedies. In a diluted state, and taken in small quantities, it excites every part of the system, renders the pulse full, communicates additional energy to the muscles, and gives temporary exaltation to the mental faculties. In some states of acute disease, characterized by excessive debility, it is a valuable remedy. In the form of brandy it is frequently given in the sinking stages of Typhus with advantage. Other kinds of ardent spirits are occasionally administered, and each is supposed to have its peculiar qualities. Thus, according to Dr. Paris, brandy may be esteemed simply cordial and stomachic; rum, heating and sudorific; and gin and whiskey, diuretic. Physicians ought to be on their guard not to prescribe alcoholic remedies in chronic diseases, whether alone or in the form of tincture, for fear of begetting habits of intemperance in their patients. Externally alcohol is sometimes applied to produce cold by evaporation, or to stimulate when evaporation is repressed."

Now, from this enumeration of the properties of the subject in question, I apprehend that every unprejudiced Physician would hesitate in expunging from his list of remedies one so manifestly calculated to do good, and withal so manageable as alcohol is known to be; until he should have, at least, presumptive proof, that for every case in which this remedy has been administered with benefit, there exists some adequate article within reach as a substitute, and at the same time not likely to entail upon his patients, those evils and deplorable consequences, too permanently affecting their health, morals, and happiness.

But where, in the Materia Medica, shall we find so general an excitant of the languid system, communicating fulness to the debilitated pulse, and energy to the prostrated muscles? I ask for information, and will most cordially adopt the remedy, whenever I shall become acquainted with it.

Is *Opium* the substitute? Its virtues, which have been appreciated from the days of Hippocrates, seem calculated to answer all the indications for which alcohol is administered, and in addition, by its direct action on the nervous system, has a powerful effect in diminishing sensibility, irritability, and mobility. It also induces tranquil and refreshing sleep, or at least drowsiness, which evidently recruits the wasted energies of the patient. It is thus a sedative, in the proper sense of the term, judging by intent, purpose, and effect. Independently of this last important and highly sanative property, opium may be made to act as a stimulant, more certain and universal than alcohol in any of its modes.

Yet, numerous as are the benefits to be derived from the proper *use* of opium, it is undeniable that evils of magnitude result from its *abuse*, not only as a narcotic *poison*, taken or administered *as such;* but also by its *habitual* use, as an allayer of pain, a reliever of spasm, and a direct tranquilizer of the troubled mind, in which last respect, though not so immorally injurious, it is more extensively so than alcohol, being resorted to by many in the higher grades of society, as well as among the indigently wretched, and with little risk of exposure for perhaps a long series of years.

It is, however, in my opinion, demonstrable, that the *juvantia* derivable from this article, do far exceed the *lædentia*—and that therefore Practitioners will very reluctantly, if at all, consent to deprive themselves of it. But may not the *alcoholic* menstrua of opium be altogether superceeded by the *aqueous* and *acid?* It is not improbable that decoctions of theP oppy, together with the preparations of Morphia, in which its narcotic principle is said to reside, especially in composition with decoctions and essential Oils of plants of the order of *Piperitæ*, and with rubefacients, may eventually enable us to dispense with alcohol in every form.

It may have been discovered that I view opium as the chief supplanter of alcohol; but notwithstanding it may be in this respect, *ipse agmen;* yet it does remain a desideratum to obviate, or neutralize, some of its potent mischiefs, by sedulously selecting from our therapeutic treasury, on all occasions which may authorize us, those lesser agents, which, either singly, or in concert, may effect our wishes: for example,

As *Antispasmodics*, we have Musk, Castor, Ammonia, Asafœtida, Galbanum, Valerian, &c.

Among the *Diaphoretics*, are, Camphor, Ipecacuan, Ammonia, Calomel, Sarsaparilla, Serpentaria, Salvia, Zingiber, and many others.

The *Rubafacients*, are, Sinapi. Capsicum, Zingber, Pix Burgundica, Spt. Terebinth. Ol. Monard, Punctat.

As *Soporifics*, Hyosciamus, Datura Stramonium, Humulus Lupulus, &c.

For *Stimulants*, there are Peppermint, Herb Mastich, Sweet Majoram, Pennyroyal, Thyme, Monarda P.

For *Sedatives*, Cold Water, *plaintive Music*, &c.

For *Astringents*, Galls, Pomegranate, Rhatany, Catechu, St. John's Wort, and others.

The above classification seems to comprise articles having all the properties, for which both alcohol and opium are celebrated. I know not but that some may smile at the introduction of *Music* into the list of remedies; but I am persuaded that it merits a high rank among them, as capable of "ministering to a mind diseased;" and had I the control of a *Hospital for the insane*, I should think myself culpable, if I omitted a special provision for the introduction and scientific administration of that almost supernatural agent.

EDWIN A. AT LEE.

*Philadelphia, February*, 1834.

